# Humans are urged to be vigilant against spillback infection of new henipaviruses

**DOI:** 10.3389/fimmu.2022.1035456

**Published:** 2022-10-03

**Authors:** Hao Yuan, Yuanni Shi, Xiaofan Chen, Jingbo Zhai, Jin Zhang, Zi-Guo Yuan

**Affiliations:** ^1^ College of Veterinary Medicine, South China Agricultural University, Guangzhou, China; ^2^ Guangdong Provincial Key Laboratory of Zoonosis Prevention and Control, College of Veterinary Medicine, South China Agricultural University, Guangzhou, China; ^3^ Medical College, Inner Mongolia Minzu University, Tongliao, China; ^4^ Key Laboratory of Zoonose Prevention and Control at Universities of Inner Mongolia Autonomous Region, Tongliao, China; ^5^ Department of Pharmacology and Toxicology, University of Mississippi Medical Center, Jackson, MS, United States

**Keywords:** humans, spillback, infection, henipaviruses, transmission

Langya henipavirus (LayV), the discovery of a novel animal-derived henipavirus in Shandong and Henan, China. First reported on August 4, 2022 (1). Research shows 35 patients with acute LayV infection, 26 patients with fever, fatigue, cough, anorexia, myalgia, nausea, headache, and vomiting, and abnormalities of thrombocytopenia, leukopenia, and impaired liver and kidney function. There were no death cases. Among 25 species of small wild animals surveyed, LayV RNA was predominantly detected in shrews, indicating that they may be a natural host of LayV.

Hendra virus and Nipah virus are emerging zoonotic viruses and members of the genus henipavirus spp. (Paramyxoviridae Family) (2). Hendra virus caused infection in horses and humans in Australia, first reported in 1994. Nipah virus-induced infection in pigs and humans in Malaysia and Singapore was first reported in 1998 (3). The natural hosts of both viruses are fruit bats (Pteropus Family) and rodents. Diseases caused by henipavirus are an important emerging disease that can cause zoonotic in the Asia-Pacific region (4). In addition, statistics show that the mortality rate of human infection with henipavirus is 40-75%. The mortality rate may also be related to the level of local sanitary condition care and treatment ability (5, 6). LayV is a newly discovered virus in China, however, it has some similarities to henipavirus in terms of transmission route and clinical symptoms. Main transmission pathway is respiratory droplet, and transmission can also occur through contact with the throat or nasal secretions of diseased wildlife, or if contact is made with the tissues of diseased wildlife (7, 8).

In recent years, there have been many emerging infectious diseases, often transmission between humans has a high threat to human society, for example, MERS and SARS-CoV-2. The big concern is that the spillover of LayV infection from animals to humans can easily pass between people, triggering a global pandemic like COVID-19. Based on the above reports, the investigation of the infected people has no contact history between them, and in the cases, no significant aggregation in space or time, so there is no evidence of transmission between humans. Regrettably, humans pay little attention to the severity of spillback to animals compared with the research on spillover infection to humans. As we all know, human metapneumovirus (hMPV), a respiratory pathogen, generally only causes influenza-like infection in humans. However, some of the endangered mountain gorillas caused by hMPV infections were dead in succession from 2009 to 2020 (9). The spillback may not only cause “secondary spillover” and endanger animal and human health but also the wild animal populations (especially endangered species) and humans (groups with important public health significance) affected by the spillback may face catastrophic risks.

For SARS-CoV-2 viruses, no spillback from white-tailed deer to humans was observed in recent research (10). However, studies on wild white-tailed deer infected with various SARS-CoV-2 variants have shown that the SARS-CoV-2 virus may survive in wild white-tailed deer for a long time, bringing new opportunities for them the further evolution of novel coronavirus and its spillover transmission to humans.

So far, there is no targeted vaccine or effective treatment for henipavirus. The only treatment is controlled therapy to manage complications. Therefore, in the case of suspected cases, isolation should be implemented as soon as possible, and infection control measures should be taken, reducing contact with wildlife, and increasing disease surveillance and prevention for wildlife. Meanwhile, due to the changes in climate and land industrialization, the habitat of thousands of mammals will be changed, which will promote the spread of viruses among different mammals. Special attention should be paid to the spillover infection after the infected human population with LayV. Therefore, the monitoring of LayV should be combined with animal movement and biodiversity investigation as soon as possible in areas where new viral diseases occur, and the climate is rapidly warming.

## Author contributions

HY, YS, XC, and Z-GY are responsible for wirting the draft. Z-GY is responsible for the conception and polishing the MS. JBZ and JZ are responsible for revising the draft. All authors contributed to the article and approved the submitted version.

## Funding

This work was supported by the National Natural Science Foundation of China (31972707) and Guangdong Provincial Forestry Department’s Provincial Financial Special Fund for Ecological Forestry Construction-Wildlife Conservation.

## Conflict of interest

The authors declare that the research was conducted in the absence of any commercial or financial relationships that could be construed as a potential conflict of interest.

## Publisher’s note

All claims expressed in this article are solely those of the authors and do not necessarily represent those of their affiliated organizations, or those of the publisher, the editors and the reviewers. Any product that may be evaluated in this article, or claim that may be made by its manufacturer, is not guaranteed or endorsed by the publisher.
